# A Composite Hydrogel Containing Mesoporous Silica Nanoparticles Loaded With *Artemisia argyi* Extract for Improving Chronic Wound Healing

**DOI:** 10.3389/fbioe.2022.825339

**Published:** 2022-03-25

**Authors:** Leyi Xue, Tewei Deng, Rui Guo, Lu Peng, Junjun Guo, Fang Tang, Jingxia Lin, Sufang Jiang, Huijuan Lu, Xusheng Liu, Lili Deng

**Affiliations:** ^1^ The Second Clinical College of Guangzhou University of Chinese Medicine, Guangzhou, China; ^2^ The Second Affiliated Hospital of Guangzhou University of Chinese Medicine (Guangdong Provincial Hospital of Traditional Chinese Medicine), Guangzhou, China; ^3^ Key Laboratory of Biomaterials of Guangdong Higher Education Institutes, Guangdong Provincial Engineering and Technological Research Centre for Drug Carrier Development, Department of Biomedical Engineering, Jinan University, Guangzhou, China; ^4^ School of Nursing Hunan University of Chinese Medicine, Changsha, China; ^5^ State Key Laboratory of Dampness Syndrome of Chinese Medicine, The Second Affiliated Hospital of Guangzhou University of Chinese Medicine, Guangzhou, China

**Keywords:** *Artemisia argyi* extract (AE), mesoporous silica nanoparticle (MSN), methacrylate gelatin (GelMA), methacrylate hyaluronic acid (HAMA), chronic wounds

## Abstract

Chronic wounds are a major health problem with increasing global prevalence, which endangers the physical and mental health of those affected and is a heavy burden to healthcare providers. *Artemisia argyi* extract (AE) has excellent antibacterial and anti-inflammatory properties. In this research, we developed AE loaded composite hydrogel scaffold based on methacrylate gelatin (GelMA)/methacrylate hyaluronic acid (HAMA) and mesoporous silica nanoparticle (MSN) as sustained-release drug carrier vehicles for the treatment of chronic wounds. The presented GelMA/1%HAMA hydrogel possessed stable rheological properties, suitable mechanical properties, appropriate biodegradability, swelling, sustained-release AE capacity. *In vitro* antibacterial and cell experiments showed that the GelMA/HAMA/MSN@AE hydrogel had excellent antibacterial activity and biocompatibility and induced macrophages to differentiate into M2 phenotype. *In vivo* wound healing of rat full-thickness cutaneous wounds further demonstrated that the prepared GelMA/HAMA/MSN@AE hydrogel could significantly promote chronic wound healing by upregulating the expression of IL-4, TGF-β1, CD31, and α-SMA but downregulating the expression of TNF-α and IFN-γ and promoting M1-M2 macrophages polarization. Altogether, we believe that the GelMA/HAMA/MSN@AE hydrogel will have wide application prospects in healing chronic wounds.

## Introduction

Chronic wounds have been regarded as a “silent epidemic,” presenting a major challenge for the healthcare system (Ferriol and Moran, 2021), among which the most common ones are varicose ulcers, diabetic foot ulcers, and pressure ulcers (Deptuła et al., 2021; Osi et al., 2021). The prevalence of chronic diseases has also increased annually, especially in developed countries, as a result of the improvement in quality of life and the increase in risk factors such as diabetes and obesity (Milho et al., 2019). In addition, due to the progress in healthcare, the continuous improvement of the survival rate of the population and the emergence of an aging population, the incidence and prevalence of chronic wounds will continue to rise in the coming decades (Wang et al., 2019).

The impact of chronic nonhealing wounds is enormous, resulting in prolongation of hospitalization and economic burden on patients with the use of expensive wound care products, which further increases the burden on the national healthcare system (Jennings et al., 2019; Rodrigues et al., 2019). Meanwhile, it also endangers the physical and mental health of patients, even leading to amputation or sepsis, increasing the disability rate, and reducing their quality of life (Liu et al., 2020). In addition, patients may feel self-blame for the wound and feel powerless for the prognosis, but positive and correct treatment can show the hope of healing (Kapp et al., 2018). Additionally, the possibility of wound healing is related to wound duration. The possibility of successfully healing the wound will significantly reduce with the extension of the time of wound appearance, which will increase the difficulty of wound healing (Borda et al., 2018). Therefore, the demand for wound treatment and care is increasing. The effective treatments of promoting wound healing and reducing the risk of wound infection and inflammation are still urgently required in chronic infectious wounds.

As a commonly used Chinese herbal medicine, *Artemisia argyi* has been widely used in promoting wound healing. The active compounds from *Artemisia argyi* leaves can easily permeate the skin and have the effects of activating blood circulation, transforming qi, dispelling dampness, dissipating cold, eliminating swelling, resolving blood stasis, relieving pain, and promoting necrotic tissue shedding (Hou et al., 2019). Moreover, *Artemisia argyi* extract (AE) has a variety of pharmacological activities, such as anti-allergic, anticoagulant, complement activation, anti-inflammatory, antibacterial, antiviral, and sedation, which can reduce tissue edema, promote granulation growth for wound tissue, and avoid adverse consequences of long-term antibiotic treatment (Bao et al., 2013; Yun et al., 2016; Wang et al., 2019; Yang et al., 2020). However, there are some limitations of AE application, such as poor aqueous solubility and easy volatilization, resulting in low bioavailability and thus making its application difficult in practice. Prior study has been demonstrated that mesoporous silica nanoparticles (MSN) are excellent candidates for drug loading owing to their good biocompatibility, biochemical and physicochemical stability, large specific surface area and pore volume, and strong loading capacity, which can improve the low bioavailability of drugs (Wang et al., 2021).

Recently, natural hydrogels have been applied to the field of biomedicine due to unique physical and biological characteristics, which are similar to the extracellular matrix (Elkhoury et al., 2021). Meanwhile, hydrogels not only can be used as drug release carriers of antimicrobial substances but also kill bacteria through their inherent antimicrobial properties (Feng et al., 2021). As an inexpensive and easily obtained natural biomaterial, gelatin can be obtained by partial hydrolysis of collagen. It has been widely used in 3D bioprinting and biomaterials (Yue et al., 2017; Garcia-Cruz et al., 2021), especially in repairing skin tissue. However, pure methacrylated gelatin (GelMA) hydrogel presents low mechanical modulus, and thermal stability is relatively poor. Therefore, we added methacrylated hyaluronic acid (HAMA) to form an interpenetrating polymer network (IPN) to improve the viscosity and maintain its gel stability at higher temperatures (Schuurman et al., 2013).

The principal purpose of this study was to prepare a suitable hydrogel system carrying AE to treat chronic wounds, which have antibacterial properties, can promote M2 macrophage polarization, and enhance collagen deposition and angiogenesis, thus effectively promoting wound healing. In this study, GelMA and HAMA were synthesized, characterized, and then mixed with MSN-loaded AE. The GelMA/HAMA/MSN@AE hydrogel was prepared under an ultraviolet (UV) light condition. The porous structure, physical properties, drug release, and antibacterial properties of this hydrogel were characterized. The slow and sustained release of AE in the hydrogel can be realized by a large specific surface area and large pore volume of MSN. In addition, the cytocompatibility in hydrogels was studied in detail by CCK-8 staining and living/dead cell staining. Moreover, an excellent therapeutic effect for promoting the repair of skin defects was shown in *in vivo* experiments. In summary, all of these results demonstrated that the GelMA/HAMA/MSN@AE hydrogel could improve patients’ feeling of medication, prolong the efficacy time of AE, and facilitate chronic wound healing.

## Experimental Section

### Materials

*Artemisia argyi* extract (AE) was purchased from Anhui Chinature Biological Co., Ltd. (Anhui, China). Gelatin (Gel, derived from cold water fish skin, adhesive strength ∼500 g bloom) was obtained from the Sigma-Aldrich Chemical Company (Shanghai, China). Hyaluronic acid (HA, Mw = 4–10 kDa) and methacrylic anhydride (MA) were purchased from Macklin Biochemical Technology Co., Ltd. (Shanghai, China). Phenyl-2,4,6-trimethylbenzoylphosphinate (LAP) was obtained from Yinchang New Material Co., Ltd. (Shanghai, China). Live/dead cell staining kits and Cell Counting Kit-8 (CCK-8) were obtained from BestBio Co., Ltd. (Shanghai, China). The bacteria strains of *Escherichia coli* (*E. coli*, ATCC-8739) and *Staphylococcus aureus* (*S. aureus*, ATCC-14458) were obtained from Luwei microbial Sci&Tech Co., Ltd. (Shanghai, China). Dulbecco’s Modified Eagle Medium (DMEM) and Fetal Bovine Serum (FBS) were obtained from Gibco (Waltham, MA, United States).

### Synthesis of Methacrylated Gelatin (GelMA)

GelMA was prepared according to the previous method (Zandi et al., 2021; Zhang et al., 2021). Briefly, 20 g of gelatin was dissolved in 250 ml ultrapure water at 60°C. Then, 12 ml of MA was added dropwise into the gelatin solution. After stirring 12 h at 37°C, the solution was dialyzed in a dialysis bag (3,500 Da molecular weight cutoff) against ultrapure water for 3 days. Then, the solution was freeze-dried to obtain GelMA polymer and stored at −20°C for future use.

### Synthesis of Methacrylated Hyaluronic Acid (HAMA)

HAMA was prepared as previously described with slight modification (Fan et al., 2020; Chen et al., 2021). Briefly, 1 g of HA was stirred in 100 ml of ultra-pure water until completely dissolved. Then, 3 ml of MA was added dropwise into the hyaluronic acid solution and stirred for 8 h at room temperature (pH was kept at about 8.5 by adding 5 mol/L sodium hydroxide). Finally, the reaction mixture was dialyzed in a dialysis bag (MWCO 8–14 kDa) against ultra-pure water for 3 days. The resulting solution was freeze-dried to attain HAMA polymer and stored at −20°C for further use.

### Synthesis of Mesoporous Silica Nanoparticle (MSN)

Uniform-sized MSN was synthesized according to a previously reported method (Wu et al., 2018; Xie et al., 2019). Totally, 2 g of hexadecyl trimethyl ammonium chloride (CTAC) and 0.07 g of triethanolamine (TEA) were dissolved in 20 ml ultra-pure water under vigorous stirring at 95°C. After the solution stabilized for 1 h, 1.5 ml of tetraethyl orthosilicate (TEOS) was dropped into the resulting solution within 2 min, and the reaction continued to stir for 1 h at 95°C. The resulting MSN was collected by centrifuged at 15,000 r/min for 5 min and washed three times with ethanol to remove the residual reactants. The final sample was dialyzed in a dialysis bag (MWCO 3500 Da) against 1% (wt%) NaCl/methanol solution for 3 h to remove the template agent CTAC and centrifuged at 15,000 r/min. Morphological analysis of MSN was visualized under a transmission electron microscope (TEM, Zeiss LIBRA 200 FEG, Oberkochen, Germany).

In order to prepare AE-loaded MSN (MSN@AE), 10 mg of AE and 50 mg MSN were added into 5 ml of 25% ethanol solution and stirred for 24 h at 37°C. The mixture was centrifuged, and the AE concentrations of residues in the supernatant were measured by a UV spectrophotometer to determine the MSN loading capacity (UV-5200, Metash Instruments, Shanghai, China).

### Preparation of Composite Hydrogels

The freeze-dried GelMA and HAMA were dissolved in PBS at 60°C to make the final GelMA concentrations of 10% (w/v) with different HAMA concentrations of 0.5% (w/v), 1% (w/v), and 2% (w/v), named GelMA, GelMA/0.5%HAMA, GelMA/1%HAMA, and GelMA/2%HAMA, respectively. Then, 0.1% (w/v) photo-initiator LAP was added, and the prepolymer solution then exposed to the UV light (365 nm) for 30 s. For the preparation of GelMA/HAMA/MSN and GelMA/HAMA/MSN@AE, the MSN 0.5% (w/v) and MSN@AE 0.5% (w/v) were added indirectly into above GelMA10% (w/v)/HAMA 2% (w/v) mixed solutions, respectively.

### Characterization of Polymers

The chemical structure of GelMA and HAMA were characterized by ^1^H NMR using a nuclear magnetic resonance spectrometer (DR×500, Bruker, Germany). Fourier transform infrared spectroscopy (FTIR) spectra of Gel, GelMA, MSN, MSN/AE, GelMA/HAMA/MSN, and GelMA/HAMA/MSN@AE were measured by Fourier transform infrared spectrometer (Spectrum One, Perkin Elmer, Norwalk, United States).

The surface area, pore size, and pore volume of MSN and MSN/AE were measured by the BET method using Micromeritics ASAP2460 instrument (Norcross, GE, United States). The micromorphology of the composite hydrogels was analyzed by scanning electron microscopy (SEM; S-3400, Hitachi, Japan). The average pore size of hydrogels was calculated by Nanomeasure software. Thermal gravimetric analysis (TGA) measurements of MSN, MSN/AE, GelMA/HAMA/MSN, and GelMA/HAMA/MSN@AE were performed with a TGA thermogravimetric analyzer (Netzsch Instruments, Selb, Germany).

### Physical Properties of Hydrogels

#### Swelling Ratio

The swelling ratio of the composite hydrogel was investigated using a gravimetric method (Osi et al., 2021). The test hydrogels were placed in PBS solution. At given time points, the samples were weighed after wiping off the surface excess water by a weighing paper. The swelling ratio of hydrogels was calculated according to the following formula:

$$Swelling\;ratio\;=\;\left(W_{t}-W_{0}\right)/W_{0}\times 100\%,$$

where W_t_ and W_0_ mean the weight of hydrogel at times t and 0, respectively.

### Porosity

The hydrogel was soaked in PBS for 24 h to reach swelling equilibrium and then freeze-dried. The volume (V) of the freeze-dried hydrogel was accurately measured with a Vernier caliper. The mass of the dry sample (W_1_) and the mass of the dry sample immersed in anhydrous ethanol (density = ρ) for 2 h (W_2_) were weighed. The porosity (P) was calculated according to the following formula:

$$P\left(\%\right)=\frac{W_{2}-W_{1}}{\rho \times V}\times 100.$$

### Rheological Measurements

Dynamic strain scanning was carried out with a TA rheometer instrument (MCR 301, Graz, Austria). The change curves of the storage modulus (G′) and loss modulus (G″) of the hydrogels were detected. In order to assess the shear viscosity behavior, the flow sweep assay was tested with a shear frequency range of 0.1–10 Hz at room temperature. Moreover, the viscoelastic region with a fixed frequency of 1 Hz was recorded over time performed at a strain of 1%.

### Compression Test

The compression modulus of GelMA/HAMA hydrogels was recorded by a universal testing machine (ELF3200, Bose, United States). The 500 μL hydrogels were prepared as a cylindrical shape (height = 6 mm and diameter = 12 mm). The compressive strain rate was fixed at 0.05 mm/s, and the strain level reached 60% of the maximum. Meanwhile, Young’s modulus could be calculated as the slope at the initial linear region.

#### *In Vitro* Biodegradation

The 300 μL hydrogel samples were immersed in a PBS solution containing either 0 or 1000 U/ml lysozyme at 37°C. At given time points, the samples were taken out from the solution and rinsed three times with ultra-pure water. The dry weight of samples was weighed after freeze-drying. The weight loss ratio was calculated according to the following equation:

$$Weight\;loss\;ratio\;\left(\%\right)\;=W_{t}/W_{0}\times 100,$$

where W_t_ and W_0_ corresponded to the weight of lyophilized hydrogel at times t and 0, respectively.

### *In Vitro* Drug Release

The composite hydrogels were immersed in 10 ml PBS and incubated at 100 rpm at 37°C. At given time points, 1 ml supernatant was collected and replaced with 1 ml fresh PBS. The AE concentration was measured using a UV spectrophotometer at λ = 345 nm. The concentration of AE released from hydrogels was back-calculated using a standard curve.

### *In Vitro* Antibacterial Behavior

Gram-negative (*E. coli*) and Gram-positive (*S. aureus*) were cultured in a sterilized LB liquid medium and diluted to an optical density of 600 nm of 0.1. Then, 400 μL tested hydrogels were prepared in a 48-well plate. Each sample was cocultured with 1.8 ml bacterial suspension and incubated at 37°C for 4 h under constant shaking. The resulting bacterial liquid (100 μL) was plated on an LB agar plate after a series of dilutions and incubator for 24 h. In order to determine the antibacterial activity, the antibacterial rate was determined according to the equation as follows:

$$Antibacterial\;rate\left(\%\right)=\frac{\left(N_{control}-N_{sample}\right)}{N_{control}}\times 100,$$

where N_control_ and N_sample_ are the numbers of bacterial colonies of the GelMA/HAMA hydrogel sample and the hydrogel with different concentrations of AE, respectively.

### Assessment of Cytocompatibility

Mouse fibroblast cells (L929 cells) were cultured in DMEM supplemented with 10% FBS, 100 μg/ml streptomycin, and 100 U/ml penicillin at 37°C in an incubator containing 5% CO_2_.

The CCK-8 and live/dead cell staining methods were used to assess the cytotoxicity of hydrogels. Briefly, 500 μL hydrogels were prepared in 48-well plates and irradiated by UV light for 30 s. Then, 400 μL cell suspension (1 × 10^4^ cells/ml) was added to the hydrogel surface. After incubation for 1, 2, and 3 days, each well was immersed in 300 µL CCK-8 and incubated in a 5% CO_2_ humidified incubator at 37°C for 1 h. The absorbance of each well was measured at 450 nm using a microplate reader (SH1000, Corona, Japan).

For the live/dead staining, each well was added 100 µL live/dead stock solution and incubated for 20 min. Cells were observed under an inverted fluorescence microscope (Olympus FV3000, Nikon, Japan). Meanwhile, the tested hydrogels were fixed to evaluate L929 cells morphology. TRITC Phalloidin was used to counterstain cytoskeleton, and diamidino-2-phenylindole (DAPI) was used to counterstain cell nuclei.

### The Transformative Effect on Macrophage Phenotype

The RAW 264.7 cells were seeded in a 6-well plate containing DMEM at densities of 1 × 10^4^ cells/well and cultivated overnight at 37°C in an incubator containing 5% CO_2_ for later use. Secondly, the sterilized hydrogel was immersed in the DMEM (containing LPS); Then, the hydrogel extract was collected after culturing at 37°C for 24 h and cocultured with cells for 48 h. The sample without the hydrogel extract was used as the control group. Characterization of M1/M2-polarized macrophages was determined by the WB technique. Raw 264.7 macrophages were lysed using RIPA lysis buffer supplemented with 1% PMSF on ice for 20 min and subjected to Western blot analysis. Each protein sample was subjected to electrophorese by 10% sodium dodecyl sulphate-polyacrylamide gel (SDS-PAGE). Then, the protein was transferred to polyvinylidene difluoride (PVDF) membrane, further incubated with different primary antibodies, anti-rabbit iNOS (Abcam, Ab178945, 1: 1,000), and anti-rabbit CD206 (Novus NBP1-90020, 1: 1,000), overnight at 4°C and treated with horseradish peroxidase- (HRP-) conjugated secondary antibodies for 1 h at 22°C. Blots were developed using an enhanced chemiluminescent reagent (Beyotime, Jiangsu, China), and the signals were detected *via* X-ray films. The expression levels of protein were quantified by IPP 6.0 software.

### Evaluation of *In Vivo* Wound Healing

#### Rat Wound Model

All animal experimental protocols have been reviewed and approved by the Animal Protection and Use Committee of Jinan University. Sprague Dawley female rats, 200–250 g, were used in this study. Rats were anesthetized by 3% pentobarbital *via* intraperitoneal injection (45–60 mg/kg) prior to surgery. The backs of rats were depilated with a depilatory cream and disinfected with iodophor. Four 12 mm diameter circular full-thickness skin injuries were created on the dorsum of rats by excising the dorsum with a distance of 2 cm. Then, the wound was coated with gauze, GelMA/HAMA hydrogel (200 μL), GelMA/HAMA/MSN@AE hydrogel (200 μL), commercially available hydrocolloid dressing, respectively. The hydrogels were fixed by Tegaderm (3M) to cover and protect the wound area. Sterile medical cotton gauze was served as the control group. Following surgery, each rat was housed in a single cage, and the wound hydrogels were replaced every 3 days.

#### Wound Closure Evaluation

The physical appearance was photographed, and the area of wounds was calculated using the IPP 6.0 software on days 3, 7, 10, and 14 after surgery. The wound closure rate was expressed as the percentage values of the day 0 measurements. The wound tissues, including the wound site and unwounded area, were collected for the following experiment.

### Histological Examination

Each wound tissue was isolated and fixed in 4% paraformaldehyde solution for 24 h, then, dehydrated through a graded series of ethanol, transferred into dimethylbenzene, and embedded in paraffin. Serial sections (4 μm thick) were cut and stained with hematoxylin and eosin (H&E) and Masson’s trichrome staining. Images of the stained sections were captured by the microtome (RM 2016, Leica, Shanghai, China).

### Immunofluorescence

After paraffin sections were rehydrated, slices were incubated in an antigen retrieval solution and blocking serum. Then, the primary antibodies, α-SMA (Servicebio, GB13044, 1:300), CD31 (Servicebio, GB113151, 1: 1,000), transforming growth factor-β_1_ (TGF-β_1_, Servicebio, GB13028, 1: 200), tumor necrosis factor-α (TNF-α, Servicebio, GB13452, 1: 200), interleukin-4 (IL-4, Bioss, bs-0581r, 1: 200), interferon-γ (IFN-γ, Proteintech, 15365-1-AP, 1: 2000), iNOS (Proteintech, 18985-I-AP, 1: 1,000), and CD206 (Novus, NBP1-90020, 1: 1,000), were added at 4°C overnight. Next, these sections were incubated with HRP-labeled goat anti-rabbit IgG secondary antibody for 50 min at room temperature. Subsequently, the sections were reacted with DAB solution after being washed in PBS, and the nuclei were counterstained with DAPI. Images of smear specimens were also collected by the inverted fluorescence microscope.

### Statistical Analysis

All quantitative experimental values from the studies were presented as means ± standard deviation. Analysis of variance (one-way ANOVA statistical test) was used to determine significant differences between two groups. A value of *p* < 0.05 was defined as statistically significant: *p < 0.05, **p < 0.01, and ***p < 0.001.

## Results and Discussion

### Characterization of the Hydrogel

We have developed a UV-cross-linked biocompatible hydrogel composed of photosensitive GelMA and HAMA. GelMA and HAMA were combined by cross-linking to improve mechanical properties and biological stability. Moreover, AE was used as an anti-inflammatory, antimicrobial agent, which was encapsulated by MSN to improve its bioavailability and loaded into the GelMA/HAMA hydrogel for application in faster healing of chronic wounds. These hydrogels also exhibited good antibacterial properties and comparable modulus to human soft tissue (Chen, 2017; Chen et al., 2017).

**FIGURE 1:**
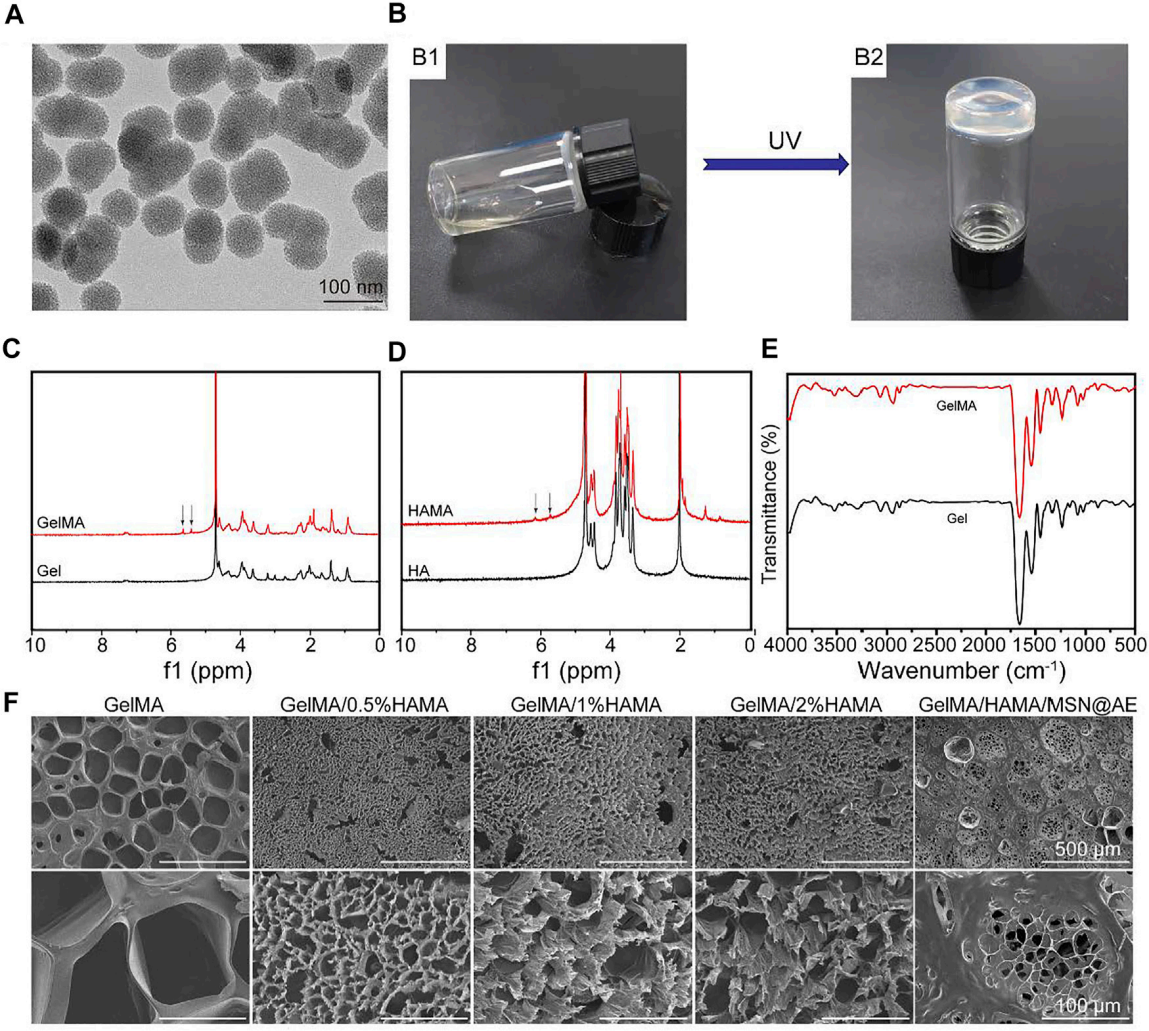
**(A)** TEM images of MSN. **(B)** Photograph of the GelMA/HAMA hydrogel through a UV cross-linking. **(C)** ^1^H NMR spectra of Gel and GelMA. **(D)** ^1^H NMR spectra of HA and HAMA. **(E)** FTIR spectra of Gel and GelMA. **(F)** SEM images of GelMA/HAMA hydrogels with different HAMA concentrations.

TEM imaging of MSN showed a uniform spherical shape with average diameters of ∼70 nm (Figure 1A). The BET analysis showed that the surface area of MSN and MSN@AE was 841.6659 and 793.2698 m^2^/g, the adsorption cumulative pore volume was 1.488,417 cm³/g and 1.377,355 cm³/g, and the adsorption average pore width was 6.9999 and 6.9297 nm, respectively. N_2_ adsorption/desorption isotherms of MSN and MSN@AE are shown in Supplementary Figure S1. As shown in Figure 1B, the synthesized GelMA/HAMA mixture hydrogel was flowing liquid before cross-linking and gradually changed into a solid phase after the UV curing. Photographs of samples containing HAMA with different concentrations are shown in Supplementary Figure S2. All the composite hydrogel is colorless and transparent, with a smooth surface.

The chemical structure of GelMA and HAMA was investigated by ^1^H-NMR. The distinctive double peaks (*δ* = 5.4 and 5.6 ppm) were observed in GelMA (Figure 1C), which verified that the gelatin had been chemically linked with methacrylate-related motifs (Yi et al., 2018). Meanwhile, the distinctive double peaks (*δ* = 5.7 and 6.1 ppm) were observed in HAMA (Figure 1D), which verified that the methacrylic group was successfully grafted onto the molecular backbone of hyaluronic acid (Eke et al., 2017).

As shown in Figure 1E, FTIR was used to characterize the presence of specific peak distribution of the infrared spectrum of polymers before and after modification. The spectrum of GelMA displayed the characteristic hydroxyl group peaks at 3,298 cm^−1^ (N-H stretching). Bands were observed at 1,656, 1,539, and 1,448 cm^−1^ belonging to the CH_2_ wagging vibrations of amide I, plane bending of N−H bond of amide II, and C−H stretching vibrations of amide III, respectively. Meanwhile, the characteristic peak intensities of amide III, amide II, and C=O in GelMA had a partial shift and change compared with gelatin, which demonstrated the newly formed amide bond. As shown in Supplementary Figure S3, the broad absorption band at 3,440 cm^−1^ is related to the stretching vibration of the OH group. The strong absorption band at 1,083 cm^−1^ is the characteristic absorption band of Si-O-Si antisymmetric stretching vibration. However, there are no other absorption peaks related to C-H, which indicates that the organic guides in the MSN have been removed at high temperatures. Therefore, FTIR showed that the composition of the MSN should be silica. There was a large wide peak in 3,500–3,700 cm^−1^, representing the characteristic absorption peak of hydroxyl in the three materials.

To further understand the thermal stability of MSN and hydrogels, we carried out their TG analysis. Meanwhile, in order to confirm the influence of *Artemisia argyi*, the TG curves of MSN and MSN@AE were almost identical (Supplementary Figure S4). Thermal degradation was carried out in three steps. The first step was related to free water loss in the hydrogel. GelMA/HAMA/MSN and GelMA/HAMA/MSN@AE hydrogels had a slight weight loss due to the loss of free water, usually below 200°C. The second step occurred between 200°C and 400°C, which was due to protein degradation. The third step occurred between 400°C and 600°C, which was due to the carbonization of the polymer material. The sample was no longer weightless after 600°C. It showed that water and other organic components had been completely volatilized, and the rest was pure silica powder.

Furthermore, SEM was conducted to visualize the changes in micromorphology of the hydrogels after freeze-drying. All hydrogels possessed a uniform porous network structure, which played an important role in the effective diffusion of nutrients and gases and provided a suitable moisture environment for the wound (Zhang et al., 2021). Figure 1F revealed that HAMA-free GelMA hydrogel consisted of large and nonhomogeneous pores, while the GelMA/HAMA hydrogels exhibited smaller and more uniform porous structures. These variations in the internal structure of the hydrogel were primarily caused by the enhancement of cross-linking degree in the GelMA/HAMA hydrogel. This three-dimensional porous structure was similar to other natural polymer hydrogel scaffolds (Chen et al., 2017), which could absorb secretions of the wound, avoiding soft tissue maceration and promoting wound healing. As shown in Supplementary Figure S5, the average pore sizes of GelMA, GelMA/0.5%HAMA, GelMA/1%HAMA, GelMA/2%HAMA, and GelMA/HAMA/MSN@AE hydrogels were 138.0 ± 20.1 μm, 14.0 ± 5.0 μm, 35.6 ± 9.4 μm, 31.8 ± 6.0 μm, and 26.1 ± 0.9 μm, respectively.

### Swelling Ratio and Porosity Assay

**FIGURE 2:**
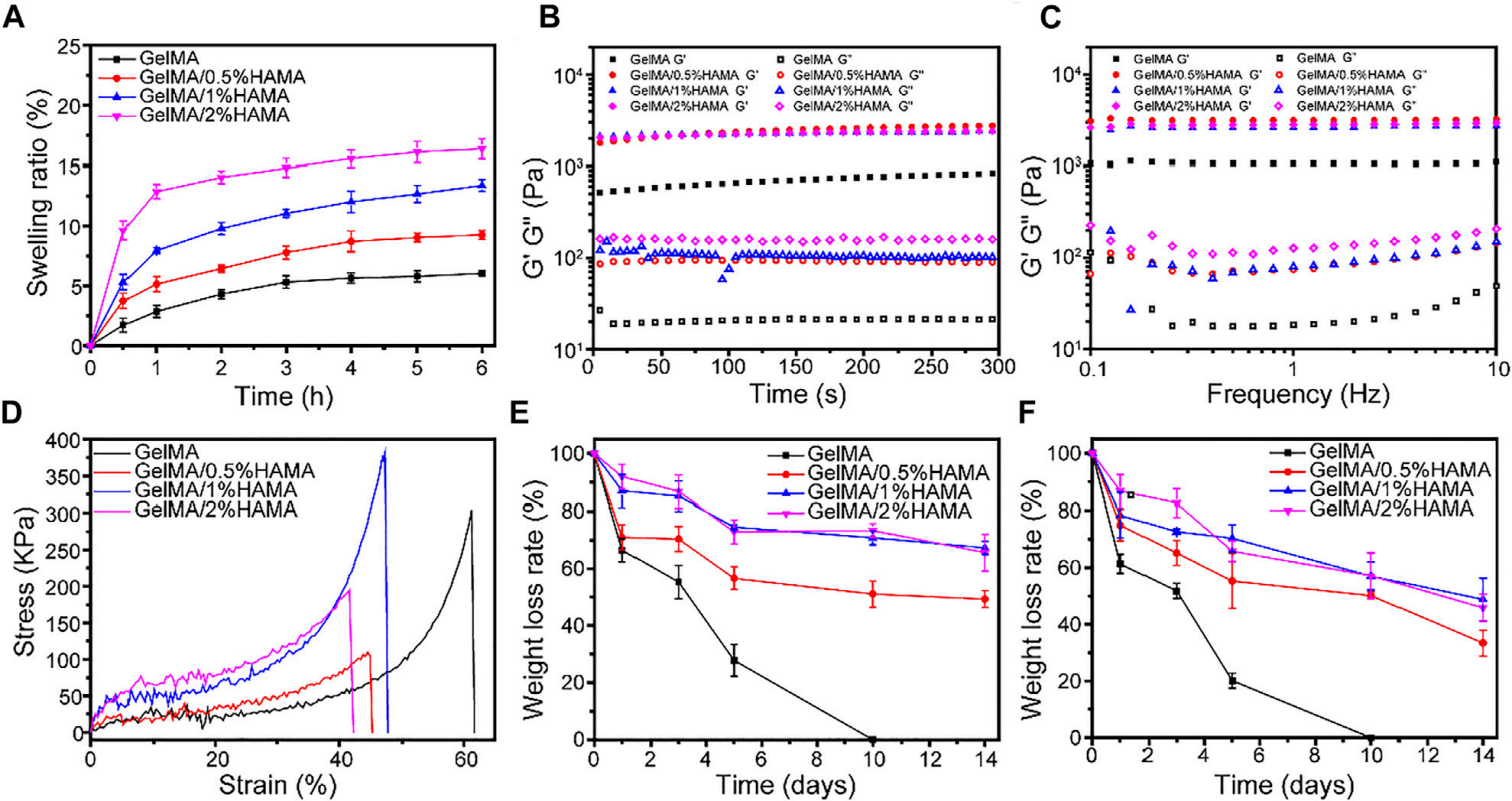
**(A)** The swelling ratio of hydrogels. **(B)** The rheological properties. **(C)** The viscosity of hydrogels with frequency ranging from 0.1 to 10 Hz. **(D)** Compressive stress-strain curves of hydrogels. **(E)** The weight loss curve of hydrogels in PBS. **(F)** The weight loss curve of hydrogels in PBS containing 1000 U/mL lysozyme.

The swelling ability of GelMA/HAMA hydrogels with different HAMA concentrations is shown in Figure 2A. The swelling ability of all hydrogels became almost saturated after 5 h, reaching their equilibrium swelling. The swelling ratios of HAMA-free GelMA, GelMA/0.5%HAMA, GelMA/1%HAMA, and GelMA/2%HAMA hydrogels were 5.7 ± 0.5%, 9.0 ± 0.4%, 12.6 ± 0.7%, and 16.0 ± 0.9%, respectively. GelMA/HAMA mixture exhibited an excellent water absorption capacity.

Usually, hydrogel dressings have excellent liquid absorption ability, which can maintain a moist wound environment and slow down the speed of liquid evaporation (Qu et al., 2018). Meanwhile, these unique characteristics have a great impact on drug release and practical applications, which endowed hydrogel with a good ability to transport nutrients and wastes (Wang et al., 2021; Yang et al., 2021). As shown in Supplementary Figure S6, the porosities of GelMA, GelMA/0.5%HAMA, GelMA/1%HAMA, and GelMA/2%HAMA hydrogels were 74.7 ± 6.1%, 80.4 ± 6.4%, 86.1 ± 5.0%, and 92.5 ± 8.8%, respectively. A prior study reported that the high porosity could provide channels for nutrient supply and metabolic exchange for internal cells (Li et al., 2016).

### Rheological and Compression Analysis

In order to evaluate the durability and integrity, the effect of HAMA concentration on the mechanical properties, the rheological properties were studied. For rheological analysis, the viscoelastic properties of hydrogels are shown in Figure 2B. When the oscillatory shear strain was fixed at 1%, it could be seen that the storage modulus (G′) of all samples was much larger than the loss modulus (G″), suggesting that the hydrogel gels rapidly. With the frequency varying from 0.1 to 10 Hz, the change of G′ was almost constant and was still larger than that of G″, indicating the elastic solid properties and good stability of hydrogels.

A wound hydrogel dressing with appropriate mechanical properties can facilitate wound healing, which is conducive to maintaining hydrogel integrity and protecting the wound from external impact. The differences of compression modulus between GelMA hydrogel with different concentrations of HAMA under compressive stress are displayed in Figure 2D. The result showed that about 60% of strain could break the GelMA hydrogel, but further increasing HAMA concentration will increase brittleness. The compression modulus of GelMA/1%HAMA hydrogel is significantly highest, up to 381.9 kPa, with about 1.3-fold higher than that of simple GelMA hydrogel, which was similar to the dermis of human skin (Lu et al., 2021). As shown in Supplementary Figure S7, Young’s moduli of GelMA, GelMA/0.5%HAMA, GelMA/1%HAMA, and GelMA/2%HAMA hydrogels were 3.3 ± 0.7, 14.5 ± 3.6, 42.7 ± 14.1, and 27.9 ± 6.8 kDa, respectively. It is seen that the GelMA/1%HAMA hydrogel has a more compact network than other hydrogels. In addition, although the failure strain of the GelMA/1%HAMA hydrogel decreased to 47%, it could still compress to 40% without breaking. In summary, 1% HAMA was selected as a suitable concentration for obtaining a composite hydrogel.

### *In Vitro* Degradation and AE Release Studies

The degradation behavior is an essential property to evaluate the ability to close tissues and support tissue regeneration (Tavafoghi et al., 2020). As shown in Figure 2E, all hydrogels exhibited obvious mass losses with increasing the incubation time. Besides, the degradation rate of samples quality in PBS without lysozyme was not as fast as in lysozyme. The GelMA hydrogel was completely degraded at 10 days. With the increase in the HAMA content, the degradation rate decreased gradually, which was likely related to the formation of a higher cross-linking-density network and denser porous structures in the hydrogel. The SEM images of freeze-dried GelMA and GelMA/HAMA hydrogels after degradation at day 3 are shown in Supplementary Figure S8. After degradation, the pore sizes of the hydrogel were increased and the porous structure was destroyed, which indicated that hydrogel was degraded with incubation. In the presence of lysozyme, the pore size of hydrogel was larger and the degradation was faster. The cracks and fragments’ porous structure of GelMA hydrogel containing 1%HAMA was less than that of pure GelMA hydrogel, which demonstrated that adding HAMA could slow down the degradation rate of hydrogel and maintain the porous structure.

The traditional ways of medication administration were directly mixing the drug with sterile gauze or hydrogel, but the encapsulated function drugs were quickly released or absorbed by gauze, resulting in the inability of drug release. Moreover, the action time of the drugs was greatly shortened, which could not meet the demand for long-term drug therapy during the process of tissue regeneration. In this study, the release curve of AE was measured to confirm whether the GelMA/HAMA hydrogel and MSN can reduce the initial burst release and realize sustained release of AE. Supplementary Figure S9A showed the standard curve of AE. Supplementary Figure S9B shows that AE from GelMA/HAMA/AE hydrogel experienced a burst release. Approximately 48.1% of the loaded AE was released at the beginning 8 h. In the following 48 h, AE was released slowly and reached a plateau, with a cumulative release of about 93.9%. In contrast, the release of AE from GelMA/HAMA/MSN@AE hydrogel was significantly prolonged. About 9.3% of AE was sustainably released at 8 h, and the AE-releasing curve was close to zero-order kinetic drug-releasing profile. These phenomena were mainly because the AE adsorbed on the outer surface and pore surface of MSN was directly dissolved into PBS without the diffusion process, resulting in a sudden release. After that, when PBS (the release medium) permeated into the MSN channels, AE diffused from the mesoporous structure of MSN to the hydrogel structure first and then escaped from the porous structure of the hydrogel, producing a concentration gradient across the surrounding fluid to achieve the sustained release of AE.

### Antibacterial Activity Evaluation

**FIGURE 3:**
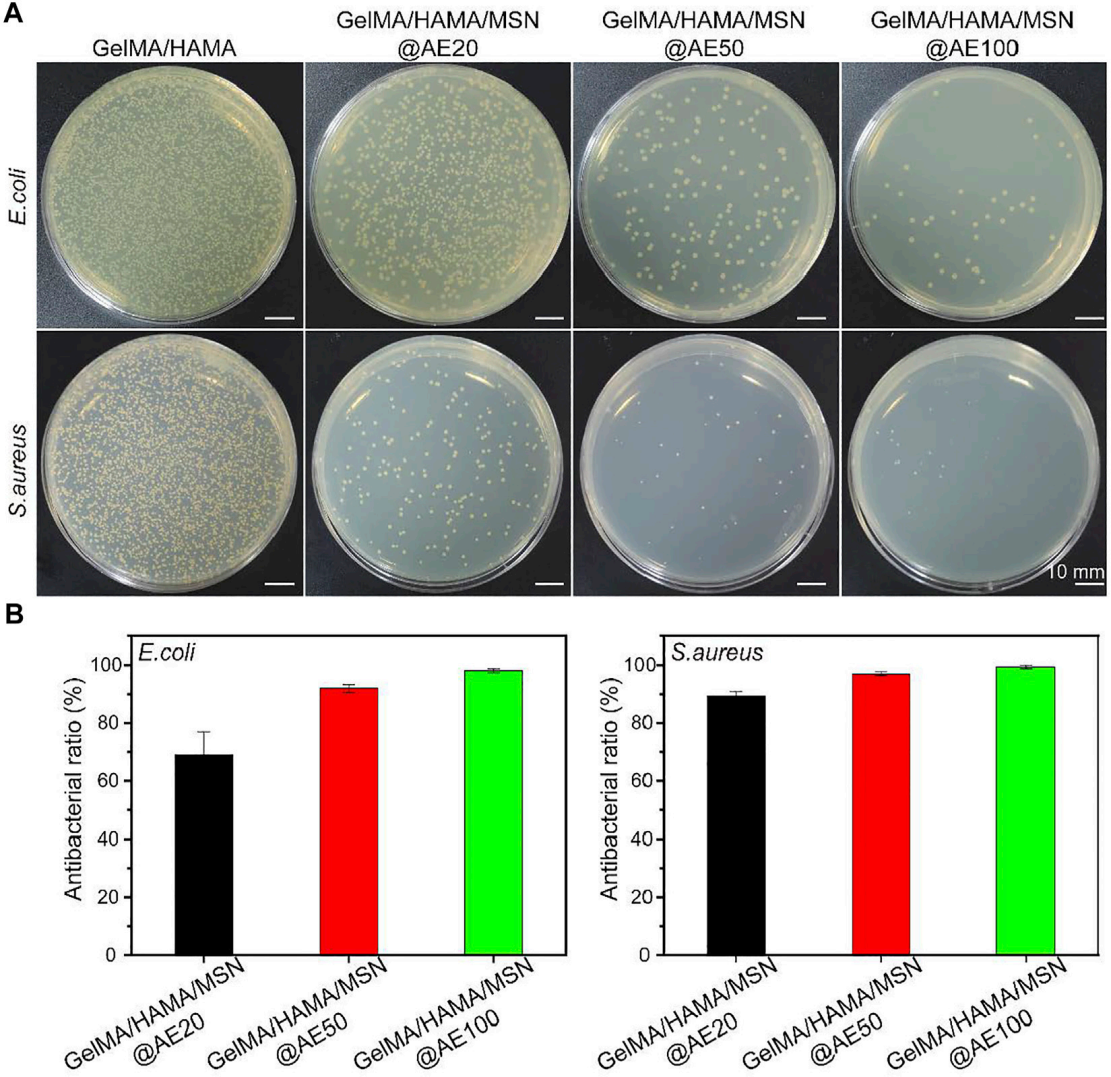
**(A)** Bacterial colonies of *E. coli* and *S. aureus* after coculture with GelMA/HAMA/MSN@AE hydrogel with different AE concentrations. **(B)** Antibacterial rate of *E. coli* and *S. aureus*.

To further evaluated the antibacterial activity of hydrogels *in vitro*, the tested hydrogels with different AE concentrations ranging from 20 to 100 μg/ml were cocultured with two bacterial strains, *S. aureus* and *E. coli*, which are involved in most infections (Huang et al., 2020; Lin et al., 2021). As shown in Figure 3A, the GelMA/HAMA/MSN@AE hydrogel exhibits good antibacterial properties compared to GelMA/HAMA hydrogels. After incubation in a medium for 4 h, the number of *S. aureus* and *E. coli* decreased significantly with the increase in AE concentration, showing a dose-dependent behavior. However, until the concentration of AE reached 50 μg/ml, its antibacterial activity increased only slightly and began to stabilize. As shown in Figure 3B, the antibacterial rates, up to 99.9 ± 0.7% of *S. aureus* and 91.9 ± 1.4% of *E. coli*, were killed by the GelMA/HAMA/MSN@AE (50) hydrogels, which demonstrated the outstanding potential for antibacterial performance of GelMA/HAMA/MSN@AE. The excellent antibacterial properties of the GelMA/HAMA/MSN@AE hydrogel were attributed to the AE, which could increase the permeability of bacterial cell membranes and inhibit the synthesis of bacterial nucleic acid, resulting in killing bacteria. We believed that GelMA/HAMA/MSN@AE had excellent antibacterial properties through the sustained release of the AE decomposing from MSN@AE, which could effectively eliminate the risk of bacterial infections in skin wounds.

### Biocompatibility of Cells in the Hydrogel

**FIGURE 4:**
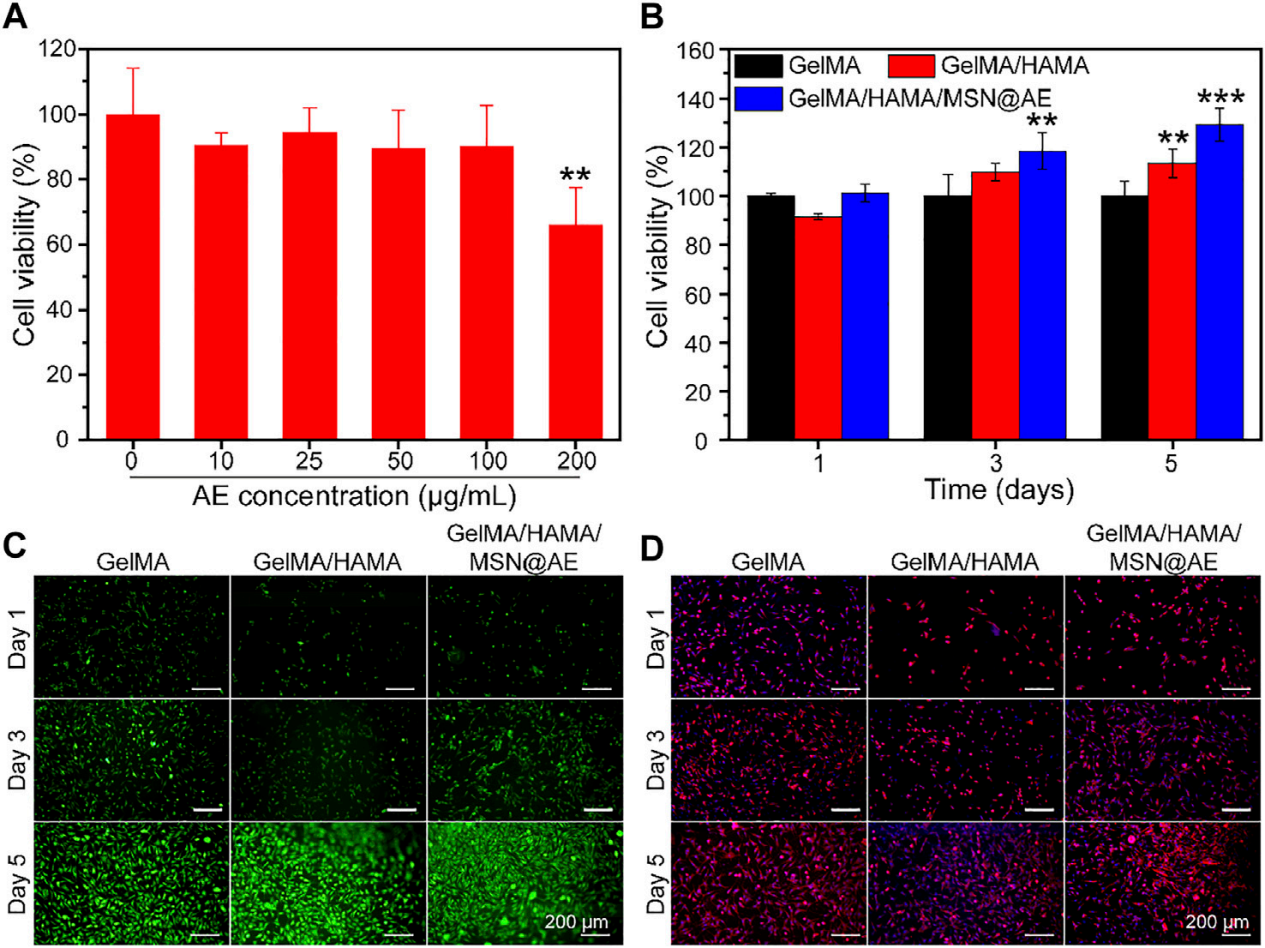
**(A)** Cell viability of MSN@AE with different AE concentrations. **(B)** Cell viability of L929 cells grown on GelMA, GelMA/HAMA, and GelMA/HAMA/MSN@AE hydrogels at days 1, 3, and 5. **(C)** Live/dead staining images of L929 cells grown on the hydrogels. **(D)** Cytoskeletal staining images of L929 cells grown on the hydrogels. (*p < 0.05, **p < 0.01, and ***p < 0.001).

The biocompatibility of composite hydrogel is a crucial factor for functional tissue regeneration and a standard for safe application in the biomedical field (Huang et al., 2021). The cytocompatibility of MSN and hydrogels was examined using CCK-8 and live/dead cell staining, respectively. As shown in Figure 4A, MSN@AE showed no cytotoxicity at AE concentrations ranging between 0 and 100 μg/ml. Based on the results of antibacterial activity evaluation and cell cytotoxicity, 50 μg/ml was selected as a suitable concentration for obtaining a composite hydrogel. As shown in Figure 4B, all hydrogel groups of cells present a high viability and proliferation activity. Especially on day 5, the GelMA/HAMA/MSN@AE group showed high cell viability (129.3 ± 6.8%), which significantly stimulated the proliferation of L929 cells. Live/dead cell staining results showed that the L929 cells seeded in GelMA/HAMA/MSN@AE hydrogel exhibited green fluorescence, indicating that most of the cells were alive (Figure 4C). As shown in Figure 4D, the morphology of L929 grown on GelMA/HAMA/MSN@AE hydrogel showed more elongation and thicker actin filaments at day 5 compared to GelMA hydrogel.

It has been recognized that the inflammatory microenvironment plays a critical role in regulating wound healing (Zhang et al., 2018). Macrophage polarization was evaluated by WB to assess the *in vitro* anti-inflammatory effect of the GelMA/HAMA/MSN@AE hydrogel. As shown in Supplementary Figures S10A,B, WB analysis showed that the expression of inducible nitric oxide synthase (iNOS), an M1 marker, was significantly downregulated in the GelMA/HAMA/MSN@AE hydrogel treated groups compared to the hydrogel-free treatment and GelMA/HAMA group, while the expression of M2 phenotype markers of anti-inflammatory macrophage (CD206) was relatively upregulated. The results demonstrated that GelMA/HAMA/MSN@AE hydrogel could help promote the resolution of the inflammatory response by facilitating the macrophages phenotype transformation from M1 to M2 and had the potential to inhibit inflammatory reactions in the process of wound healing.

### *In Vivo* Wound Healing Evaluation

#### Wound Healing Examination

**FIGURE 5:**
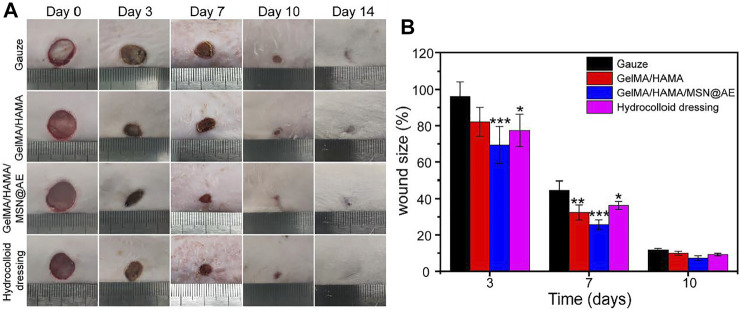
**(A)** Representative photograph of wounds treated with different materials, including Gauze, GelMA/HAMA hydrogel, GelMA/HAMA/MSN@AE hydrogel, commercially available hydrocolloid dressing at days 0, 3, 7, 10, and 14. **(B)** The changes of wound size of each group.

To further verify the clinical potential of hydrogel, a full-thickness cutaneous wound model in rats was applied to assess the wound repair capability of the GelMA/HAMA/MSN@AE hydrogel. As shown in Figure 5A, the skin defect in all groups shrank over time. Interestingly, the GelMA/HAMA hydrogel, GelMA/HAMA/MSN@AE hydrogel, and commercially available hydrocolloid groups significantly promoted wound healing, but the gauze group presented a weak effect on the acceleration of wound healing. In particular, the full-thickness cutaneous defects for the GelMA/HAMA/MSN@AE group were almost closed at day 14, followed by GelMA/HAMA and commercially available hydrocolloid groups, and the slowest in gauze group, as depicted by the quantification of the wound area in Figure 5B. The rapid wound closure may be attributed to the good skin permeability of AE percutaneous administration, which has various physiological and pharmaceutical effects, such as invigorating blood circulation, reducing swelling, relieving pain, relieving itching, and antibacterial.

On day 3, the wound size of the gauze, GelMA/HAMA hydrogel, GelMA/HAMA/MSN@AE hydrogel, and commercially available hydrocolloid were 96.1 ± 8.1%, 82.1 ± 8.0%, 69.3 ± 10.2%, and 77.4 ± 8.9%, respectively. After 7 days of operation, the wound size of the GelMA/HAMA/MSN@AE group was 25.7 ± 2.6%, which was significantly lower than that of the control group (44.6 ± 25.0%). On day 10, it can be observed that GelMA/HAMA/MSN@AE hydrogels displayed superiority in promoting wound healing and the wound was nearly closed, while the gauze group, GelMA/HAMA, and commercially available hydrocolloid group still had a wound area of 11.7 ± 0.8%, 9.9 ± 1.1%, and 9.2 ± 0.7%, respectively. In summary, GelMA/HAMA/MSN@AE hydrogels showed the smallest wound size, which was attributed to the antibacterial and anti-inflammatory properties of AE, gelatin, and hyaluronic acid’s desirable function in the promotion of wound healing, and the moist wound environment provided by the hydrogel dressing. AE could accelerate different wound healing phases (inflammation, proliferation, and remodeling), as it could effectively scavenge reactive oxygen species (ROS) and exert anti-oxidant effects during the inflammatory phase, promote fibroblast migration, collagen formation, and epithelialization in the proliferation phase, and increase the number of cytokines in the remodeling phase.

### Histological Analysis

**FIGURE 6:**
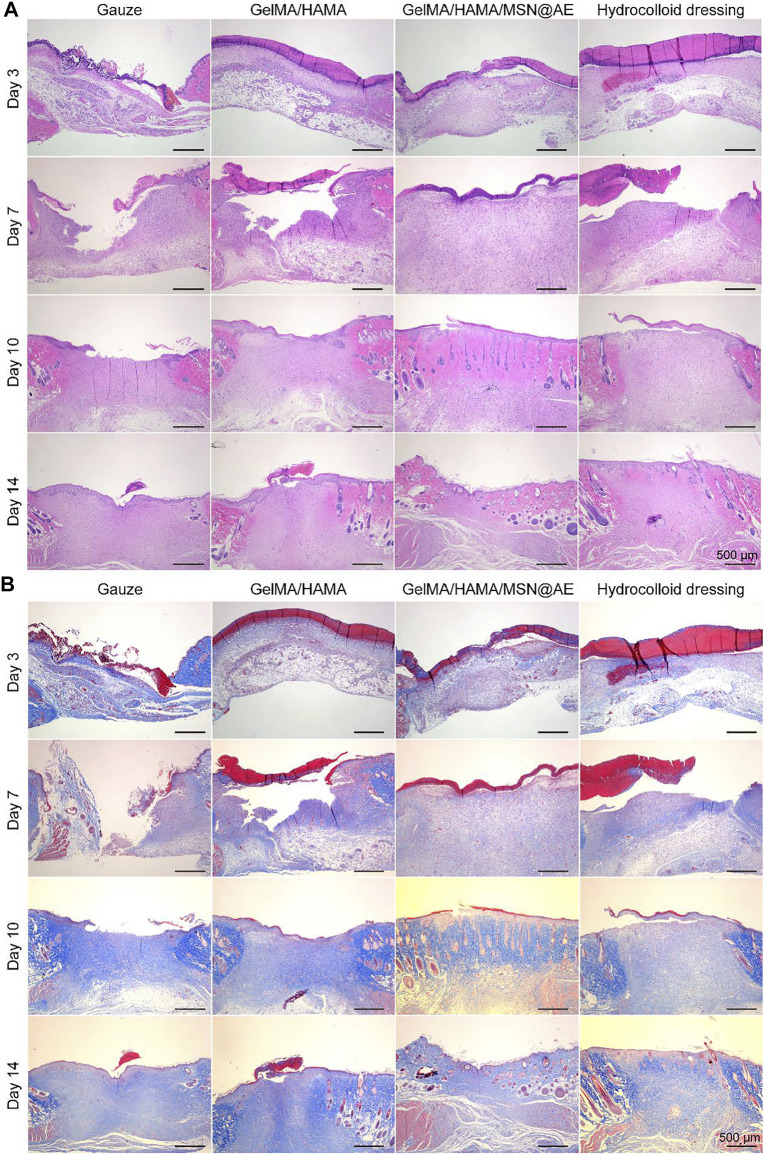
**(A)** Representative H&E staining images acquired on days 3, 7, 10, and 14 after surgery. **(B)** Representative Masson’s trichrome staining images acquired on days 3, 7, 10, and 14 after surgery.

Wound tissue regeneration and re-epithelialization is one of the evaluation criteria in the process of wound healing (Xiong et al., 2021). H&E staining was carried out to observe the morphological changes of skin layer reconstruction on days 3, 7, 10, and 14 *in vivo*. As shown in Figure 6A, a large number of inflammatory cell infiltration could be seen in gauze, while GelMA/HAMA and commercially available hydrocolloid groups showed a mild inflammatory infiltration in the GelMA/HAMA/MSN@AE group on day 3. The control group showed a large number of inflammatory cells at day 7, and the new epidermal cells were relatively few, which were still in the inflammatory stage. However, the number of inflammatory cells in the GelMA/HAMA/MSN@AE group was reduced and the epidermis was thickened at day 7, which was attributed to the well anti-oxidant action and anti-inflammation capacity of AE (Jaradat, 2021). On day 3, loose granulation tissue could be seen in the wound treated with GelMA/HAMA/MSN@AE. On day 7, granulation tissue in GelMA/HAMA/MSN@AE group became compact and thicker than that of other groups. Additionally, the treated wounds also presented a closer dermal-epidermal junction and restored the aesthetic function at day 14. What is more, intact neoepidermis could be observed in the wound tissue, while the epidermis of the control group was not evenly covered.

In addition, collagen deposition plays a significant role in the remodeling period of wound healing and is beneficial to wound contraction. Masson staining was carried out to evaluate the collagen deposition in the regenerated skin tissue. As shown in Figure 6B, the staining depth of all groups increased with time, indicating the increase in collagen content. On day 3, there were fewer collagen fibers in the control group, while the GelMA/HAMA/MSN@AE group presented more collagen fibers, and the immature collagen deposition could be seen. On day 10, the collagen fibers in GelMA/HAMA/MSN@AE group became compact and orderly, showing the basketweave arrangement of collagen bundles, while those in the control group were sparse and disordered. The arrangement characteristics of collagen fibers at day 14 in the GelMA/HAMA/MSN@AE group tended to the normal skin tissue more than other groups. Thus, the GelMA/HAMA/MSN@AE hydrogel with slow release ability of AE could facilitate wound healing through promoting re-epithelialization and collagen deposition.

### Angiogenesis Analysis

**FIGURE 7:**
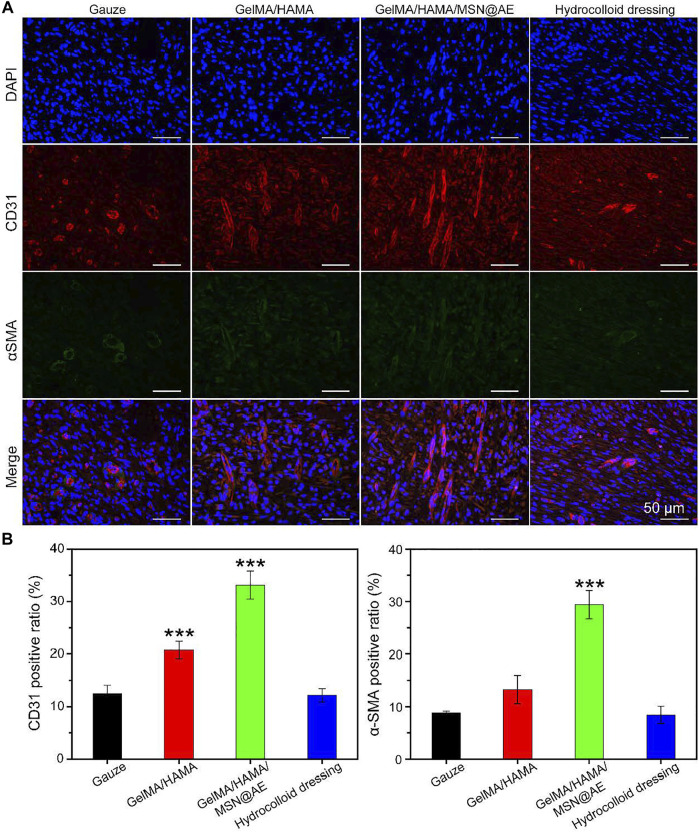
**(A)** Representative CD31/α-SMA images of immunofluorescence staining of wound sections treated with each group at day 7. **(B)** Quantitative analysis of newly formed (CD31, red) and mature blood vessels (α-SMA, green) (*p < 0.05, **p < 0.01, and ***p < 0.001).

Neovascularization is one of the key factors to promote wound healing, which reflects the degree of skin tissue regeneration and functional recovery (Yao et al., 2021). The mechanism of angiogenesis induced by GelMA/HAMA/MSN@AE hydrogel was studied by immunofluorescent staining, in which CD31 and α-smooth muscle actin (α-SMA) were the markers of neovascularization and mature blood vessels, respectively. As shown in Figures 7A,B, except for the lower density of CD31 positive vascular endothelial cells in the gauze group, the indexes of other groups increased significantly at day 7, especially in the GelMA/HAMA/MSN@AE group. In addition, the density and area of α-SMA in the subcutaneous tissue around the wound were the highest in the GelMA/HAMA/MSN@AE group, indicating that the degree of myofibroblasts activation was high in the process of wound healing (Mao et al., 2021). Thus, the GelMA/HAMA/MSN@AE group was conducted to the production of vascular endothelial cells, the synthesis of actin in the vascular wall, and promoting vascularization, which demonstrated that GelMA/HAMA/MSN@AE Hydrogel could upregulate the expression of CD31 and α-SMA and promote myofibroblasts to accelerate the wound closure.

### Inflammatory Response Analysis

Inflammation will hinder the healing process of the wound bed, but anti-inflammatory properties of AE have been unequivocally established in previous studies (Yun et al., 2016). To further explore the mechanism of hydrogel in controlling wound infection, immunofluorescence staining of four typical inflammatory cytokines, anti-inflammatory factors (IL-4 and TGF-β1), and pro-inflammatory factors (TNF-α and IFN-γ) were performed on day 3 and day 7.

**FIGURE 8:**
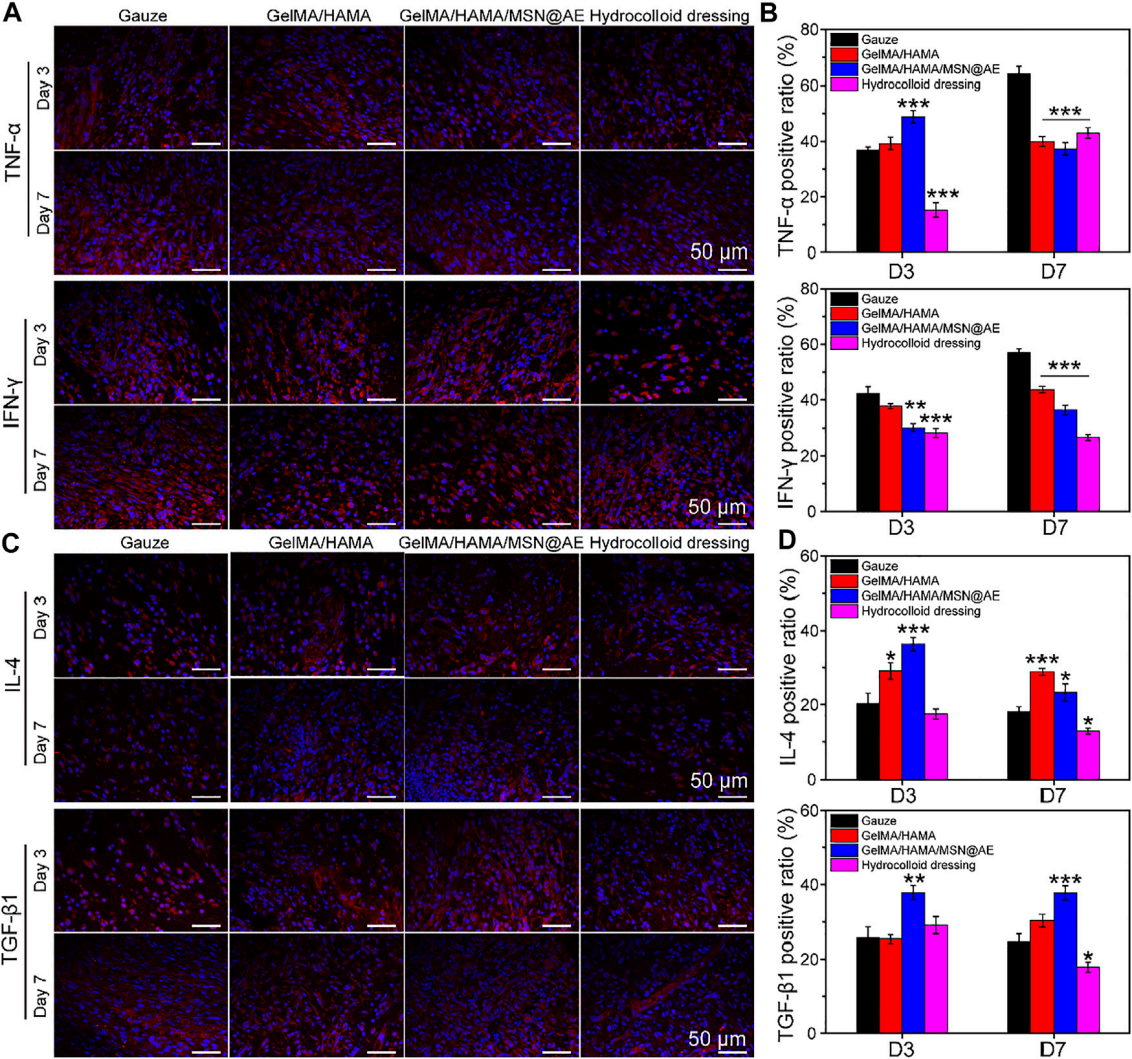
**(A)** Representative TNF-α and IFN-γ images of immunofluorescence staining of wound sections treated with each group at day 3 and day 7. **(B)** Quantitative analysis of TNF-α positive ratio and IFN-γ positive ratio. **(C)** Representative IL-4 and TGF-β1 images of immunofluorescence staining of wound sections treated with each group at day 3 and day 7. **(D)** Quantitative analysis of IL-4 positive ratio and TGF-β1 positive ratio (*p < 0.05, **p < 0.01 and ***p < 0.001).

As shown in Figures 8A,B, elevated levels of TNF-α and IFN-γ production could be monitored in each group on day 3, indicating a severe inflammatory response. However, the levels of TNF-α and IFN-γ in the GelMA/HAMA/MSN@AE group were lower than those in other groups on day 7, and a large amount of IL-4 and TGF-β1 were observed (Figures 8C,D). Meanwhile, GelMA/HAMA and commercially available hydrocolloid group also exhibited low expression compared to the control group, representing that the sustainable release of AE significantly prevented the increase in these pro-inflammatory cytokines and upregulated the expression of anti-inflammatory factors to some extent. On day 7, little expression of TNF-α and IFN-γ was seen in the GelMA/HAMA/MSN@AE group compared to other groups. Inflammation of the wounds plays an important role in the clearance of necrotic tissue and resistance to bacterial infection, thereby of a higher level of inflammation in the early stage. If the wound remains in the inflammatory stage, the wound healing rate will be delayed. The GelMA/HAMA/MSN@AE group has the immunomodulatory effect, which accelerates the transition from the inflammatory phase to the proliferation phase, thereby accelerating the wound healing process, resulting in decreased levels of TNF-α on day 7. Meanwhile, the gauze groups maintained high levels of inflammation, resulting in slower extracellular matrix deposition and slower wound healing (Larouche J et al., 2018). The statistical results revealed that GelMA/HAMA/MSN@AE hydrogel could greatly diminish the inflammation at the wound sites. All results suggested that GelMA/HAMA/MSN@AE hydrogel could upregulate the expression of IL-4 and TGF-β1 but downregulate the expression of TNF-α and IFN-γ, which exert anti-inflammatory effects.

**FIGURE 9:**
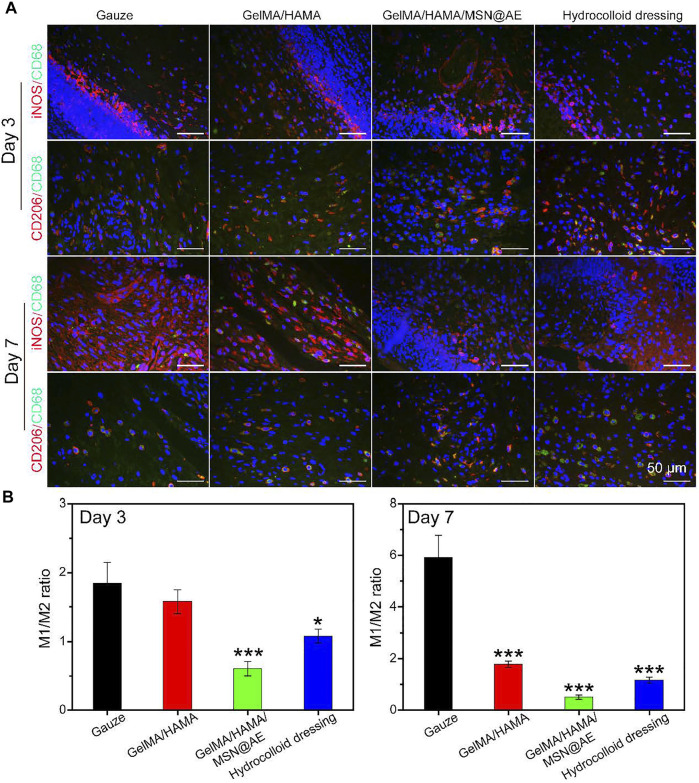
**(A)** Representative iNOS (red), CD206 (red) and CD68 (green) images of immunofluorescence staining of wound sections treated with each group at day 3 and day 7. **(B)** Ratio of M1 to M2 analysis of each group (*p < 0.05, **p < 0.01, and ***p < 0.001).

Meanwhile, macrophages can coordinate the complex processes of cell proliferation, infection, inflammation, and functional tissue regeneration in the wound (Freund et al., 2020), responding to the wound microenvironment and driving their conversion from a pro-inflammatory M1 phenotype to an anti-inflammatory M2 phenotype (Zhao et al., 2021). Hence, hydrogels with the capability of promoting M2 macrophage polarization can accelerate the progression from the inflammatory stage to the proliferative and remodeling stage of wound healing. To further elucidate the anti-inflammatory pathways of GelMA/HAMA/MSN@AE hydrogel, macrophage polarization was evaluated by immunofluorescence. As shown in Figures 9A,B, the GelMA/HAMA/MSN@AE group showed some expressions of the M2 phenotype macrophages maker (CD206), while the macrophages in the gauze group were still in the M1 phenotype, indicating the initial stage of inflammation. The inflammatory microenvironment is crucial in the wound healing phase, and the inflammatory phase of chronic wounds is severely prolonged, which fails to transition from the inflammation phase to the proliferation phase (Liu et al., 2020).

Additionally, although anti-inflammatory M2 phenotype macrophages maker (CD206) was also detected in the GelMA/HAMA and commercially available hydrocolloid groups, the GelMA/HAMA/MSN@AE group showed the most expression of CD206. All results demonstrated that GelMA/HAMA/MSN@AE hydrogel could upregulate the expression of CD206 but downregulate the expression of iNOS, which promoted the transformation of macrophages from inflammatory phenotype to a reparative phenotype and induced the wound to enter the proliferative phase. Summary, all results indicated that compared to other groups, the GelMA/HAMA/MSN@AE hydrogel group exerted anti-inflammatory effects in the chronic wound through enhanced expression of CD31, α-SMA, IL-4, and TGF-β1, reduced the expression of TNF-α and IFN-γ, and facilitated the M1-to-M2 transition of macrophages in the early stage of wound healing, playing a beneficial role in wound closure.

## Conclusion

The purpose of this work is to prepare hydrogels with antibacterial properties, immune regulation, anti-inflammation, and good biocompatibility for the treatment of wounds. Then, GelMA/HAMA hydrogel with MSN-loaded *Artemisia argyi* extract (AE) was successfully prepared. GelMA/HAMA/MSN@AE hydrogel has stable rheological properties, suitable mechanical properties, appropriate biodegradability, swelling ratio, and sustained-release AE capacity. AE could increase the permeability of bacterial cell membrane and inhibit the synthesis of bacterial nucleic acid, so GelMA/HAMA/MSN@AE hydrogel had excellent antibacterial activity. AE could regulate inflammation, and the synthetic materials of GelMA and HAMA are natural cytoplasmic matrix materials, so GelMA/HAMA/MSN@AE hydrogel had good biocompatibility and promoted M1-M2 transformation of macrophages. AE could promote the growth of collagen fibers after full-thickness skin trauma and inhibit inflammation. Hydrogel could protect the wound from external stimulation and provide a moist environment. GelMA/HAMA/MSN@AE hydrogel could increase collagen deposition, promote angiogenesis, and regulate inflammation in chronic wound healing of rats. Overall, our results concluded that GelMA/HAMA/MSN@AE hydrogel with the sustainable release ability of AE exhibited an excellent property in promoting chronic wound healing.

## Data Availability

The original contributions presented in the study are included in the article/Supplementary Material, further inquiries can be directed to the corresponding authors.
