# Supplementary material for: A Composite Hydrogel Containing Mesoporous Silica Nanoparticles Loaded With Artemisia argyi Extract for Improving Chronic Wound Healing
Source: Front Bioeng Biotechnol. 2022 Mar 25;10:825339. doi: 10.3389/fbioe.2022.825339 (PMC8990880; doi:10.3389/fbioe.2022.825339)
Supplement: Supplementary file 1 [file DataSheet1.docx]

**Supporting Information**

**A composite hydrogel containing mesoporous silica nanoparticles loaded with Artemisia argyi extract for improving chronic wound healing**

Leyi Xue ^a^, Tewei Deng ^b^, Rui Guo ^c^, Lu Peng ^b^, Junjun Guo ^b^, Fang Tang ^b^, Jingxia Lin ^b^, Sufang Jiang ^a^, Huijuan Lu ^d^, Xusheng Liu ^e, b *^, Lili Deng ^b *^

^a.^ The Second Clinical College of Guangzhou University of Chinese Medicine, Guangzhou, 510405, China

^b.^ The Second Affiliated Hospital of Guangzhou University of Chinese Medicine (Guangdong Provincial Hospital of Traditional Chinese Medicine), Guangzhou, 510120, China

^c.^ Key Laboratory of Biomaterials of Guangdong Higher Education Institutes, Guangdong Provincial Engineering and Technological Research Centre for Drug Carrier Development, Department of Biomedical Engineering, Jinan University, Guangzhou, 510632, China

^d.^ School of nursing Hunan University of Chinese Medicine, Hunan, 410000, China

^e.^ State Key Laboratory of Dampness Syndrome of Chinese Medicine, The Second Affiliated Hospital of Guangzhou University of Chinese Medicine, Guangzhou, 510120, China

^*^ Corresponding author: [liuxusheng@gzucm.edu.cn](mailto:liuxusheng@gzucm.edu.cn) (Xusheng Liu); [denglili@gzucm.edu.cn](mailto:denglili@gzucm.edu.cn) (Lili Deng).


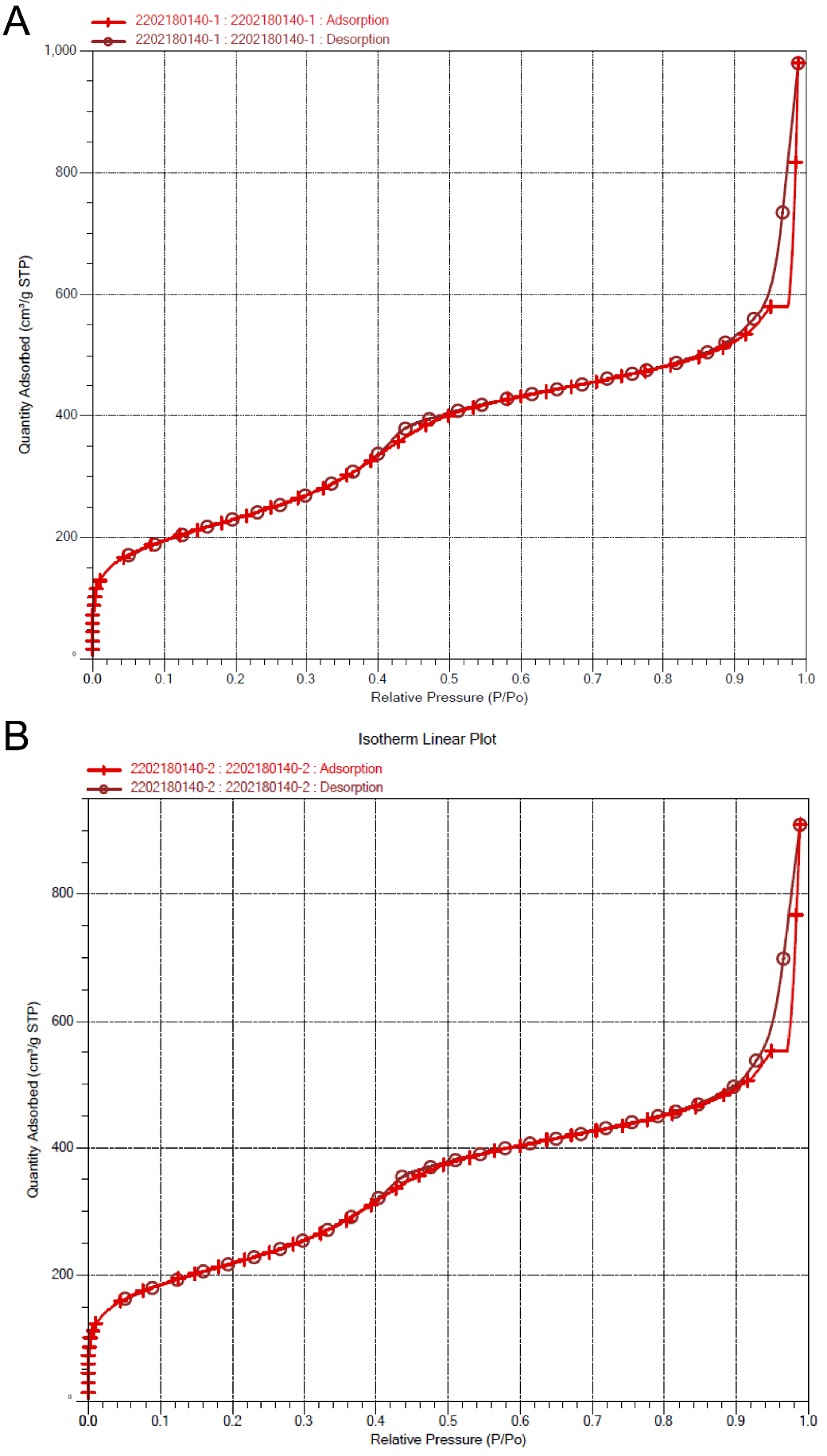


**Figure S1.** N_2_ adsorption/desorption isotherms of MSN (A) and MSN@AE (B).

**
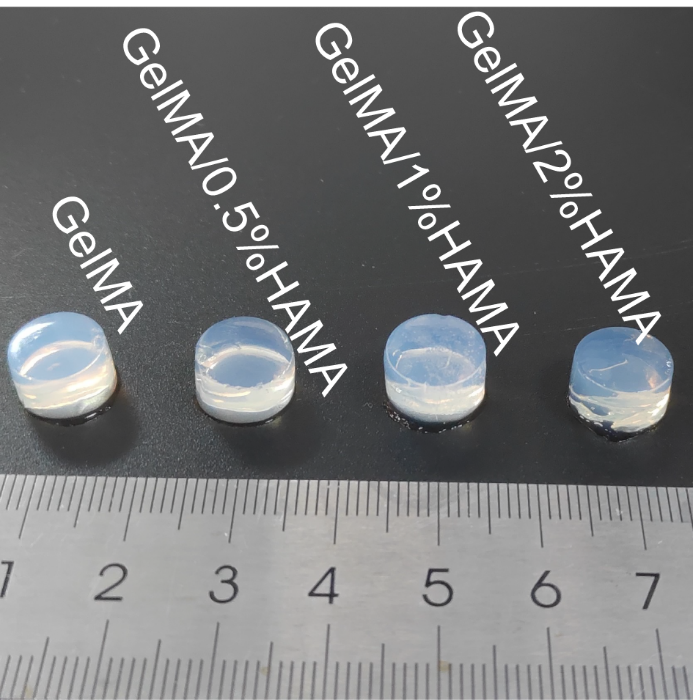
**

**Figure S2.** The cylindrical GelMA/HAMA hydrogels with different HAMA concentrations.





**Figure S3.** FTIR spectra of MSN, MSN/AE, GelMA/HAMA/MSN, GelMA/HAMA/MSN@AE.





**Figure S4.** TG curve of MSN, MSN/AE, GelMA/HAMA/MSN, GelMA/HAMA/MSN@AE.





**Figure S5.** The average pore size of GelMA, GelMA/0.5%HAMA, GelMA/1%HAMA, GelMA/2%HAMA and GelMA/HAMA/MSN@AE hydrogels.





**Figure S6.** The porosity of GelMA, GelMA/0.5%HAMA, GelMA/1%HAMA and GelMA/2%HAMA hydrogels.





**Figure S7.** The Young’s modulus of GelMA, GelMA/0.5%HAMA, GelMA/1%HAMA and GelMA/2%HAMA hydrogels.


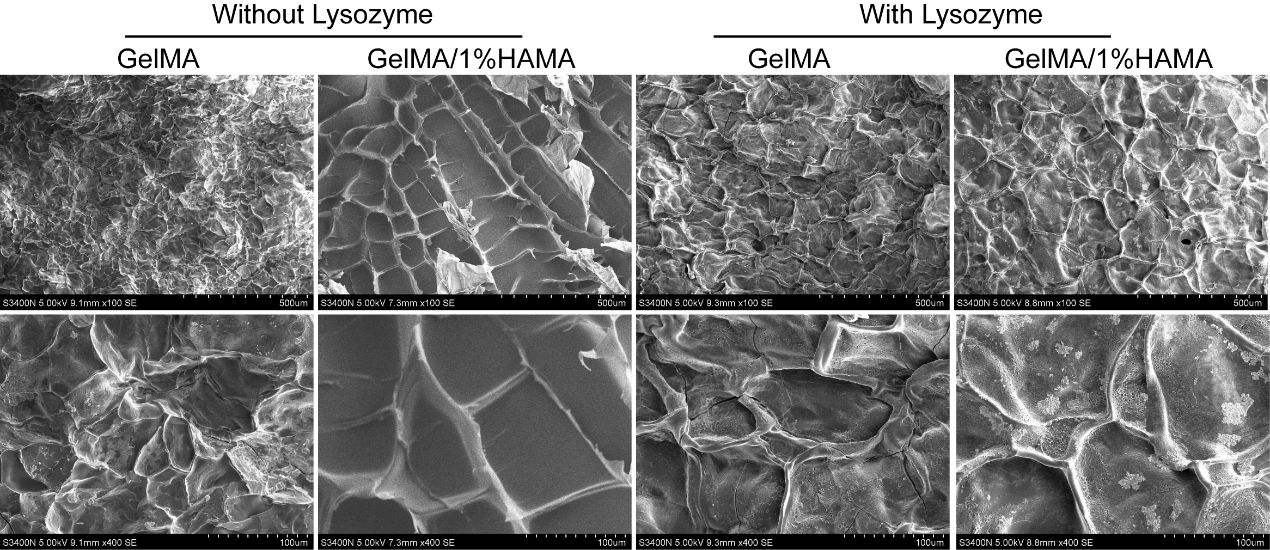


**Figure S8.** SEM images of the surface morphology of the degraded hydrogel at day 3.


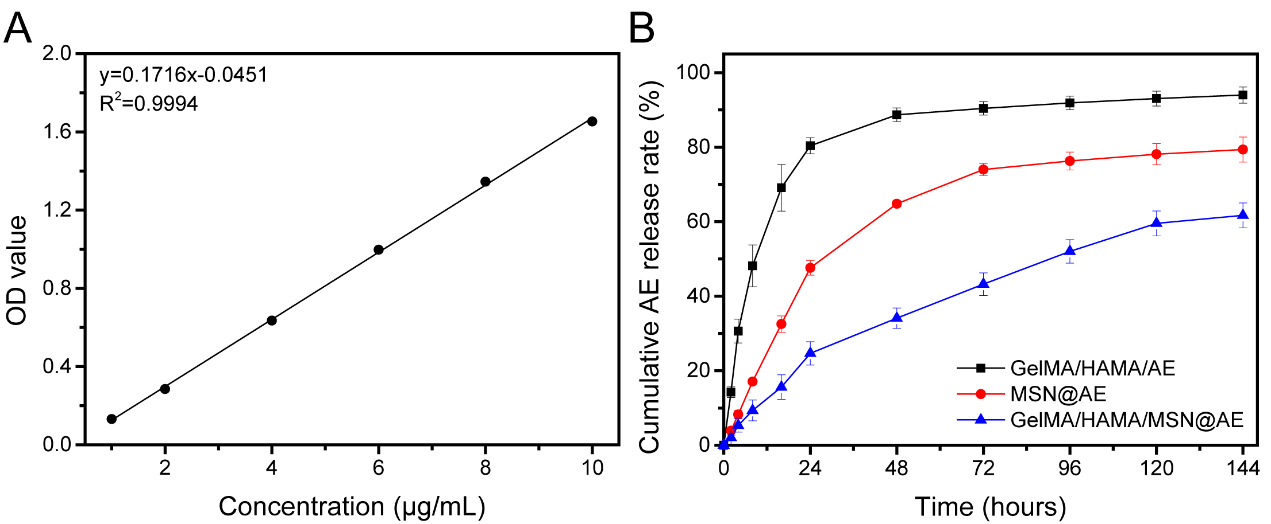


**Figure S9.** (A) The standard curve of AE. (B) Drug release curve of AE from GelMA/HAMA/AE and GelMA/HAMA/MSN/AE hydrogels.


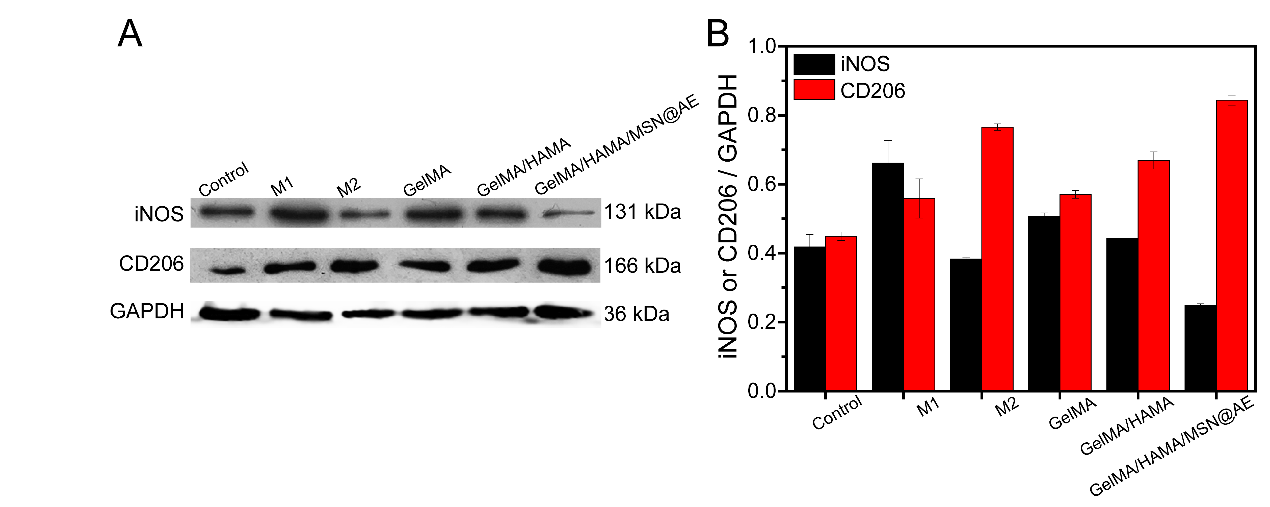


**Figure S10.** (A) Western blot analysis of macrophages polarization. (B) Quantitative analysis of WB results.
